# Supplementary material for: Estimating internationally imported cases during the early COVID-19 pandemic
Source: Nat Commun. 2021 Jan 12;12:311. doi: 10.1038/s41467-020-20219-8 (PMC7804934; doi:10.1038/s41467-020-20219-8)
Supplement: Supplementary file 1 — Supplementary Information [file 41467_2020_20219_MOESM1_ESM.pdf]

Supporting Information

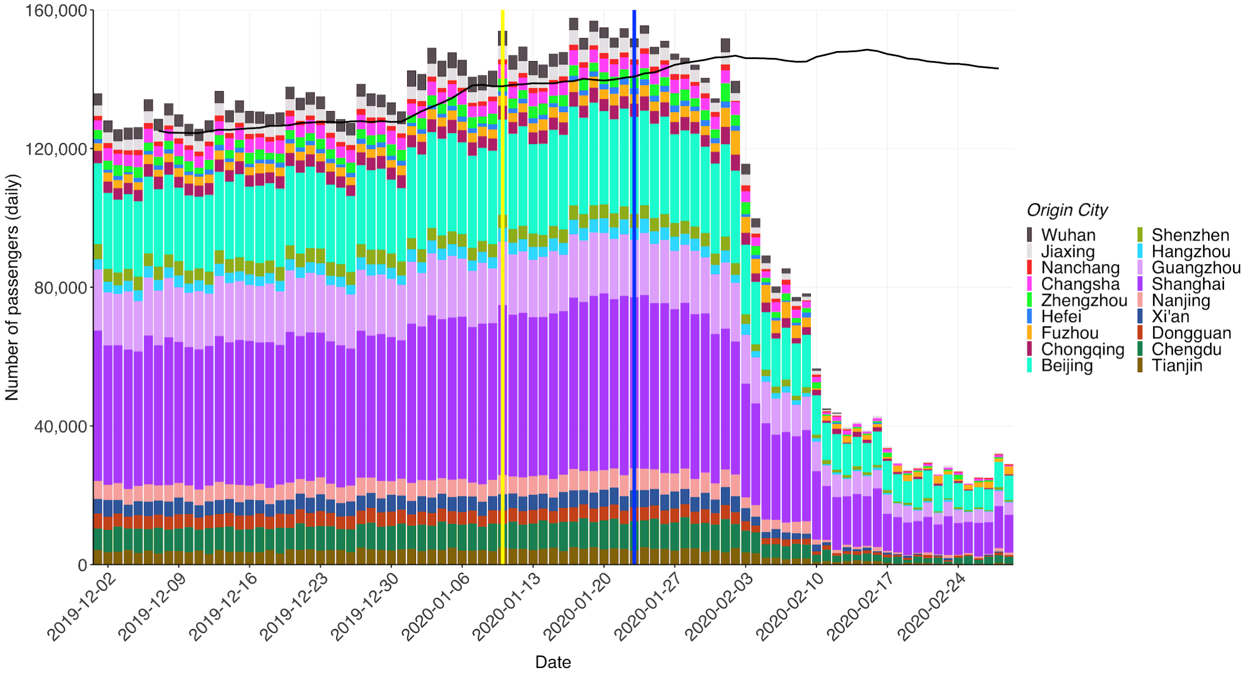

Supplementary Figure 1. Daily flight volume from Chinese cities. Estimated daily flight volume from 18 Chinese cities to 43 international destinations (10 *high-surveillance locations* for model validation, 26 African destinations, 7 additional destinations) from 1 December 2019 to 29 February 2020. The yellow vertical line marks the start of the 40-day Chunyun period surrounding Lunar New Year. The blue vertical line indicates 23 January 2020, the day travel restrictions were widely implemented in Wuhan. The curved black line shows the 7-day rolling average of the estimated daily flight volume from 18 Chinese cities to 43 international destinations for the equivalent period one year earlier: from 1 December 2018 to 28 February 2019.

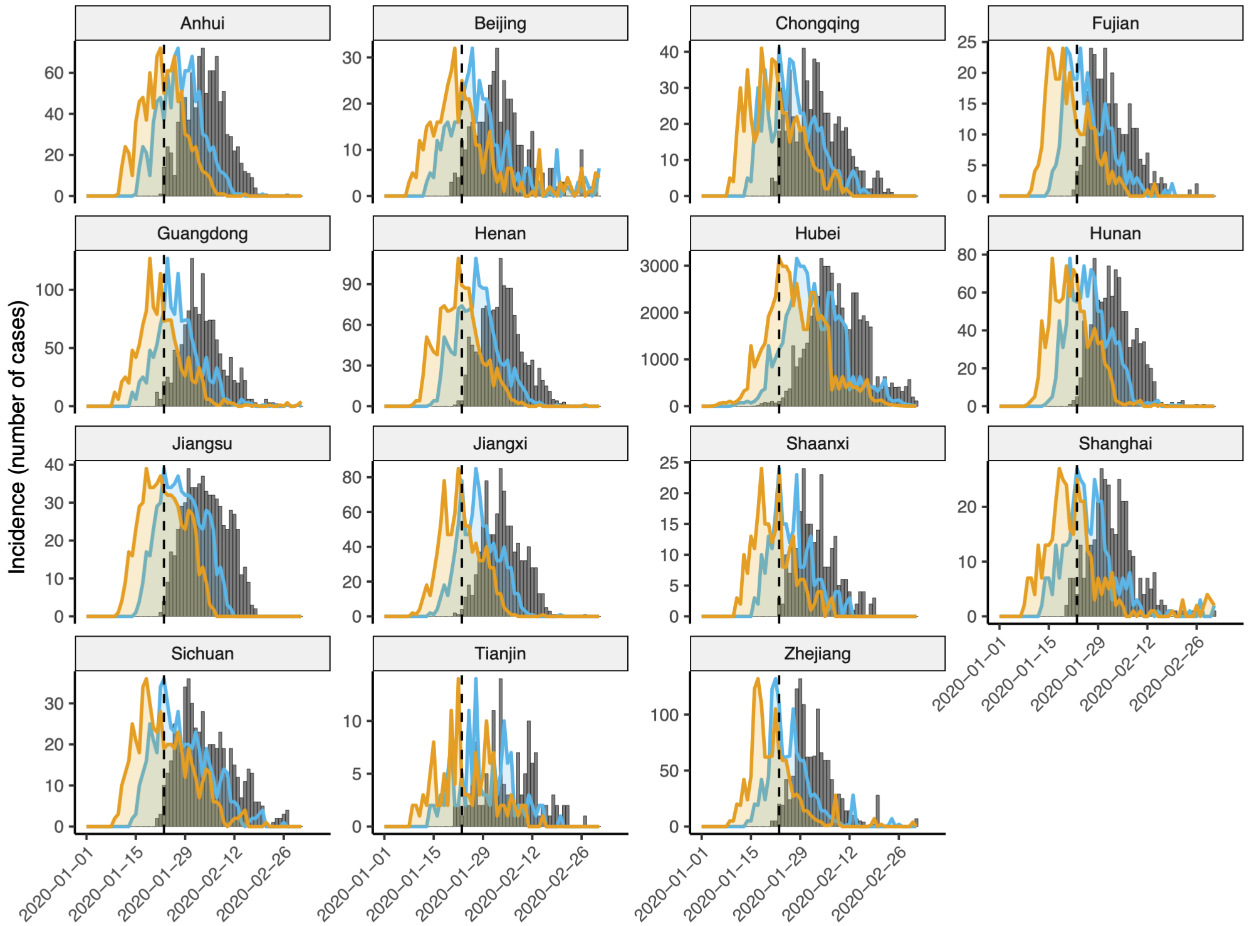

Supplementary Figure 2. Computing infections from case counts. Back-calculation of symptom onsets (blue) and infections (orange) from observed confirmed case counts (grey bars) for all provinces considered here. Confirmed cases were shifted back by 7 days (the mean confirmation delay) to estimate symptom onset incidence, then further by 5 days (the median incubation period) to estimate infection incidence of those cases. Vertical dashed line shows 23 January 2020, the date of lockdown in Wuhan.

Parameter	Scenario 1*	Scenario 2	Scenario 3	Scenario 4	Scenario 5	Scenario 6	Scenario 7	Scenario 8	Scenario 9
Ascertainment rate ratio	5:1	Time-varying	5:1	5:1	5:1	0.5:1	50:1	5:1	5:1
Days contributing to relative prevalent cases	5 days	5 days	2 days	7 days	5 days (Wuhan) and 2 days, following 3 day gap (outside of Wuhan)	5 days	5 days	5 days	5 days
Allocation of relative prevalent cases to cities	proportional to city share of reported cases	proportional to city share of reported cases	proportional to city share of reported cases	proportional to city share of reported cases	proportional to city share of reported cases	proportional to city share of reported cases	proportional to city share of reported cases	Cities hubs assumed to capture all infections	proportional to the proportion of the province population in that city

*Scenario considered most plausible (best-estimate scenario)

Supplementary Table 1. Nine model scenarios. Table gives the key parameter assumptions that we vary under our nine model scenarios.

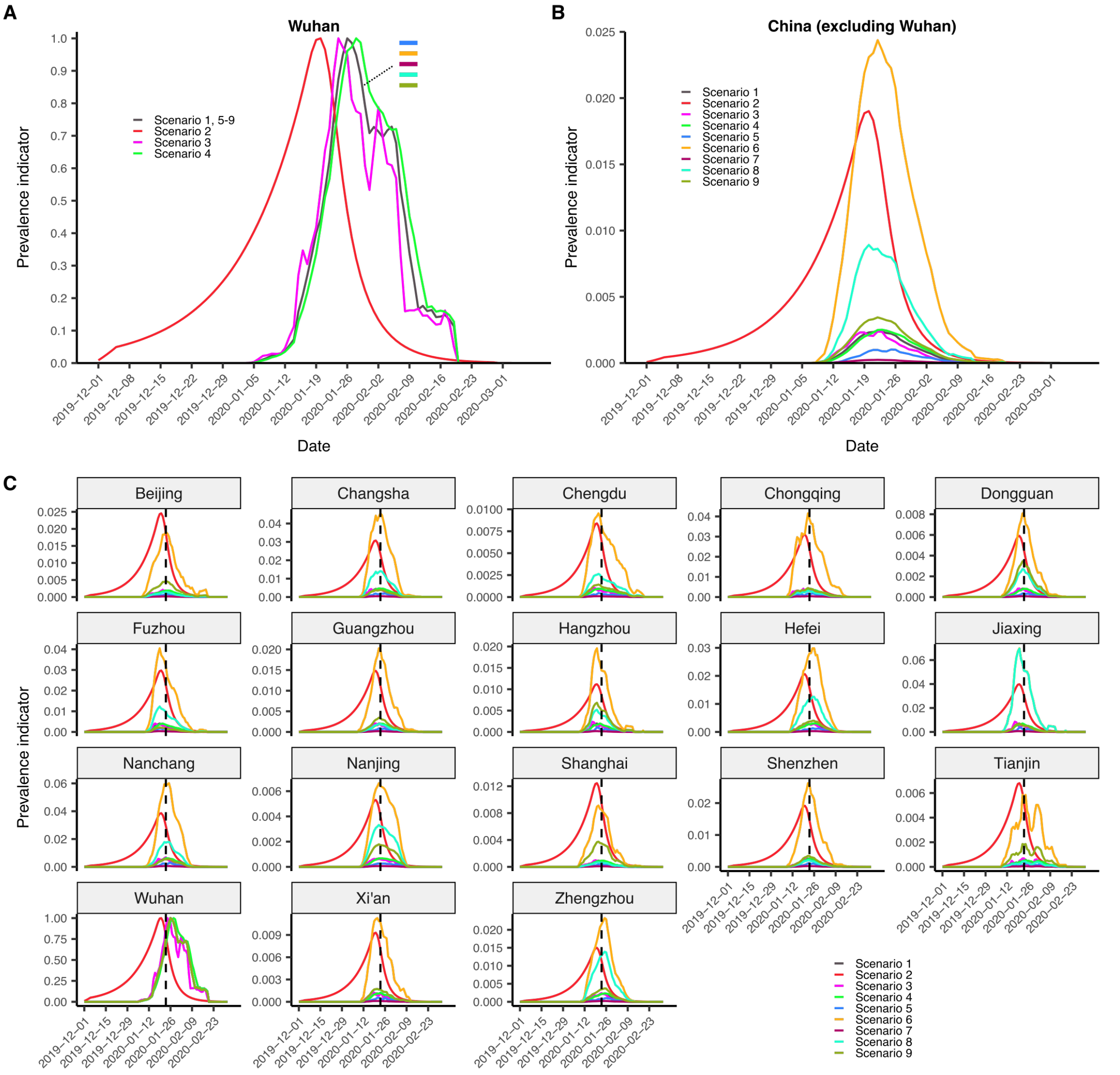

Supplementary Figure 3. Estimated prevalence indicator across China. (A) and (B) Prevalence indicator for Wuhan and averaged for non-Wuhan cities for all model scenarios. Note that only relative comparisons are meaningful, and thus all curves are scaled relative to the maximum value in Wuhan within each scenario. Note also that in (A) Scenarios 1 and 5-9 yield identical curves for Wuhan and that Scenario 2 is based on independent modeling estimates. Scenarios 1, 6 and 7 only differ in their assumed ascertainment rate ratio, which we account for in all scenarios by scaling up (or in the case of Scenario 6, scaling down) incidence in Hubei and keeping constant incidence in all other cities. Scenario 5 differs by changing the prevalence duration in non-Wuhan cities. Scenario 8 and 9 differ by the assumed distribution of province-level case counts across cities. **(C)** Prevalence indicators in the 18 considered Chinese cities. Values were scaled relative to the maximum prevalence indicator in Wuhan within each scenario. Vertical dashed line shows 23 January 2020, the date of lockdown in Wuhan.

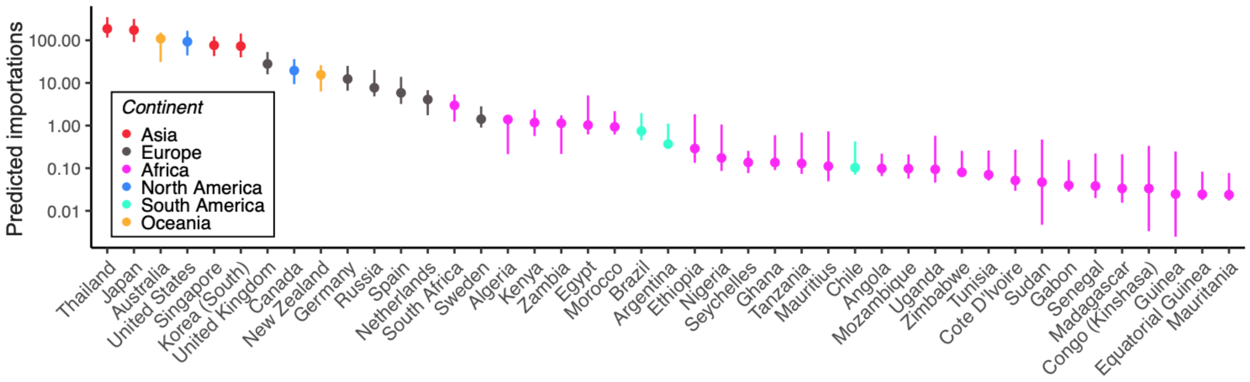

Supplementary Figure 4. Importation predictions globally. Plot showing the predicted number of COVID-19 case importations (using a log-scaled vertical axis) from China to all countries included in our analysis (Asian in red, Europe in dark brown, Africa in pink, North America in blue, South America in turquoise, Oceania in orange). Points show predictions of the best-estimate model, vertical lines indicate ranges across all model scenarios. Countries are sorted from highest to lowest number predicted cases and are colored by continent. Note that countries from each continent were selected based on having high average flight volume from China.

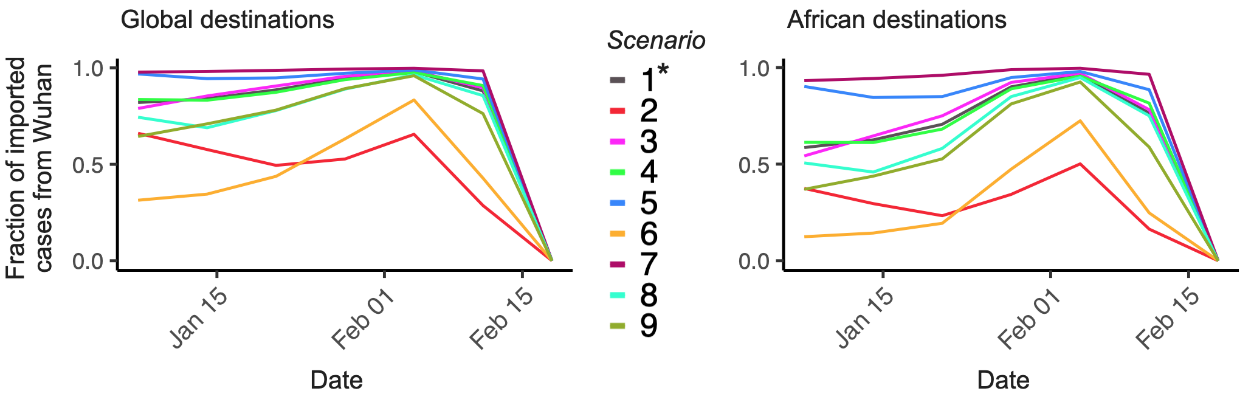

Supplementary Figure 5. Wuhan's contribution to case importations. Weekly fraction of imported cases from Wuhan to global destinations (left-hand side) and to African destinations (right-hand side) shown as mean predictions for each of the 9 scenarios. The best-estimate scenario is Scenario 1 (dark brown, highlighted with an asterisk). Other scenarios shown are Scenario 2 (in red), 3 (pink), 4 (bright green), 5 (blue), 6 (orange), 7 (dark red), 8 (turquoise), and 9 (dark green). The horizontal axis shows the date for the current year 2020.

Country	1*	2	3	4	5	6	7	8	9
**Algeria**	1.39	0.21	1.03	1.71	1.37	1.64	1.36	1.39	1.43
**Angola**	0.1	0.07	0.08	0.11	0.09	0.22	0.09	0.1	0.12
**DRC**	0.03	0.06	0.02	0.04	0.01	0.33	0	0.04	0.07
**Côte D'Ivoire**	0.05	0.07	0.04	0.06	0.04	0.27	0.03	0.05	0.08
**Egypt**	1.03	1.22	0.82	1.18	0.75	5.1	0.62	1.73	1.52
**Equatorial Guinea**	0.02	0.02	0.02	0.03	0.02	0.08	0.02	0.02	0.04
**Ethiopia**	0.29	0.39	0.22	0.35	0.19	1.84	0.13	0.29	0.51
**Gabon**	0.04	0.04	0.03	0.04	0.03	0.16	0.03	0.04	0.06
**Ghana**	0.14	0.15	0.11	0.16	0.11	0.59	0.09	0.14	0.2
**Guinea**	0.02	0.07	0.02	0.03	0.01	0.25	0	0.02	0.06
**Kenya**	1.17	0.57	0.94	1.36	1.1	2.35	1.06	1.33	1.27
**Madagascar**	0.03	0.05	0.03	0.04	0.02	0.21	0.02	0.03	0.05
**Mauritania**	0.02	0.02	0.02	0.03	0.02	0.08	0.02	0.02	0.03
**Mauritius**	0.11	0.19	0.08	0.13	0.07	0.73	0.05	0.12	0.25
**Morocco**	0.93	0.62	0.76	1.06	0.85	2.18	0.81	1.03	1.13
**Mozambique**	0.1	0.06	0.07	0.12	0.09	0.21	0.09	0.1	0.11
**Nigeria**	0.17	0.25	0.14	0.2	0.12	1.06	0.09	0.18	0.29
**Senegal**	0.04	0.06	0.03	0.05	0.03	0.22	0.02	0.04	0.07
**Seychelles**	0.14	0.08	0.11	0.15	0.13	0.26	0.12	0.14	0.16
**South Africa**	2.98	1.24	2.33	3.51	2.82	5.35	2.75	3.26	3.23
**Sudan**	0.05	0.1	0.04	0.06	0.02	0.47	0	0.09	0.09
**Tanzania**	0.13	0.17	0.11	0.15	0.09	0.69	0.07	0.15	0.2
**Tunisia**	0.07	0.06	0.05	0.08	0.06	0.26	0.05	0.09	0.1
**Uganda**	0.09	0.13	0.08	0.11	0.06	0.58	0.05	0.1	0.15
**Zambia**	1.13	0.22	0.83	1.4	1.1	1.74	1.07	1.16	1.21
**Zimbabwe**	0.08	0.07	0.07	0.09	0.07	0.26	0.06	0.09	0.11
**Total**	**10.35**	**6.19**	**8.08**	**12.25**	**9.27**	**27.13**	**8.7**	**11.75**	**12.54**

Supplementary Table 2. Table of mean imported cases to 26 African destination locations, by scenario, with our best-guess scenarios marked by an asterisk (S). Countries are ordered alphabetically.
