# Supplementary material for: Estimating internationally imported cases during the early COVID-19 pandemic
Source: Nat Commun. 2021 Jan 12;12:311. doi: 10.1038/s41467-020-20219-8 (PMC7804934; doi:10.1038/s41467-020-20219-8)
Supplement: Supplementary file 3 — Reporting Summary [file 41467_2020_20219_MOESM3_ESM.pdf]

nature research

Corresponding author(s): Rene Niehus
Last updated by author(s): Nov 11, 2020

Reporting Summary

Nature Research wishes to improve the reproducibility of the work that we publish. This form provides structure for consistency and transparency in reporting. For further information on Nature Research policies, see our Editorial Policies and the Editorial Policy Checklist.

Statistics

For all statistical analyses, confirm that the following items are present in the figure legend, table legend, main text, or Methods section.

n/a	Confirmed	
x		The exact sample size (n) for each experimental group/condition, given as a discrete number and unit of measurement
x		A statement on whether measurements were taken from distinct samples or whether the same sample was measured repeatedly
x		The statistical test(s) used AND whether they are one- or two-sided<br>*Only common tests should be described solely by name; describe more complex techniques in the Methods section.*
x		A description of all covariates tested
	x	A description of any assumptions or corrections, such as tests of normality and adjustment for multiple comparisons
	x	A full description of the statistical parameters including central tendency (e.g. means) or other basic estimates (e.g. regression coefficient) AND variation (e.g. standard deviation) or associated estimates of uncertainty (e.g. confidence intervals)
x		For null hypothesis testing, the test statistic (e.g. F, t, r) with confidence intervals, effect sizes, degrees of freedom and P value noted<br>*Give P values as exact values whenever suitable.*
x		For Bayesian analysis, information on the choice of priors and Markov chain Monte Carlo settings
x		For hierarchical and complex designs, identification of the appropriate level for tests and full reporting of outcomes
x		Estimates of effect sizes (e.g. Cohen's d, Pearson's r), indicating how they were calculated

Our web collection on statistics for biologists contains articles on many of the points above.

Software and code

Policy information about availability of computer code

Data collection	No data collection was needed. We used readily available data on COVID-19 epidemiology and we were provided data on air travel (see Data section)
Data analysis	Analysis and visualization was performed using R (version 4.0.1) and Rstudio (1.3.959). All code and data are available online [DOI 10.5281/zenodo.4266642]

For manuscripts utilizing custom algorithms or software that are central to the research but not yet described in published literature, software must be made available to editors and reviewers. We strongly encourage code deposition in a community repository (e.g. GitHub). See the Nature Research guidelines for submitting code & software for further information.

Data

Policy information about availability of data

All manuscripts must include a data availability statement. This statement should provide the following information, where applicable:
- Accession codes, unique identifiers, or web links for publicly available datasets
- A list of figures that have associated raw data
- A description of any restrictions on data availability

International Air Transport Association (IATA) data was provided by BlueDot, Toronto, Canada (https://bluedot.global). Data on departed flights was provided by Cirium (https://www.cirium.com). This data is not publicly available as raw data. Transformed flight data used for this work is freely available online (Menkir et al. 202039). COVID-19 case counts are from publicly available datasets: WHO reports (https://apps.who.int/iris/bitstream/handle/10665/331425/SITREP_COVID-19_WHOAFRO_20200311-eng.pdf) and European CDC (https://opendata.ecdc.europa.eu/covid19/casedistribution/csv), and MIDAS reports (https://midasnetwork.us/covid-19/). The derived COVID-19 prevalence data and importation risk generated in the analysis is available online (Menkir et al. 202039).

Field-specific reporting

Please select the one below that is the best fit for your research. If you are not sure, read the appropriate sections before making your selection.

- [x] Life sciences
- [ ] Behavioural & social sciences
- [ ] Ecological, evolutionary & environmental sciences

For a reference copy of the document with all sections, see nature.com/documents/nr-reporting-summary-flat.pdf

Life sciences study design

All studies must disclose on these points even when the disclosure is negative.

Sample size	This study aims to use the maximum amount of relevant data (flight data and confirmed case counts) that was to our knowledge available at the time of analysis. Thus, the amount of data used in this study is limited by pragmatic choices and the typical time-contraints when modelling ongoing disease outbreaks
Data exclusions	We did not have any data exclusion criteria
Replication	We are using observational data to make predictions for low-surveillance settings. As such, verification at this time is difficult, but we thoroughly discuss how our predictions link to observed outbreak sizes in the different locations.
Randomization	This is an observational study
Blinding	The was no blinding since this was an observational study

Reporting for specific materials, systems and methods

We require information from authors about some types of materials, experimental systems and methods used in many studies. Here, indicate whether each material, system or method listed is relevant to your study. If you are not sure if a list item applies to your research, read the appropriate section before selecting a response.

Materials & experimental systems

n/a	Involved in the study	
[x]	[ ]	Antibodies
[x]	[ ]	Eukaryotic cell lines
[x]	[ ]	Palaeontology and archaeology
[x]	[ ]	Animals and other organisms
[x]	[ ]	Human research participants
[x]	[ ]	Clinical data
[x]	[ ]	Dual use research of concern

Methods

n/a	Involved in the study	
[x]	[ ]	ChIP-seq
[x]	[ ]	Flow cytometry
[x]	[ ]	MRI-based neuroimaging
